# The Case for Limiting the Number of Offspring per Gamete Donor: A Response to Pennings

**DOI:** 10.1111/andr.70308

**Published:** 2026-07-02

**Authors:** Lucy Frith, Jackson Kirkman‐Brown

**Affiliations:** ^1^ Centre for Social Ethics and Policy Department of Law University of Manchester Manchester UK; ^2^ Birmingham Women's & Children's NHS Foundation Trust Birmingham UK; ^3^ Centre For Human Reproductive Science University of Birmingham Birmingham UK

**Keywords:** donor, quota

## Abstract

Pennings’ recent paper addresses a very current and important issue in reproductive donation, should we limit the numbers of children born from each gamete donor? In this perspective piece, we raise counter‐arguments to Pennings’ claims that the use of gamete donors should not be limited.

1

Pennings’ recent paper [1] addresses a very current and important issue in reproductive donation, should we limit the numbers of children born from each gamete donor? This is an area that the European Society of Human Reproduction and Embryology (ESHRE) is addressing. ESHRE has produced recommendations on this, chaired by the authors [2]. The *ESHRE position paper: international limits on the number of offspring per gamete donor* makes the case for an international limit on the numbers of children born from a gamete donor, and starting with a European limit, suggests that ultimately a maximum of 15 families should be created per donor. Our initial suggestion is that the number per donor is limited to 50, and moves are made to work towards the goal of 15 families. It should be stressed that even the 50‐family limit would be a significant reduction in the use of some donors, the European Sperm Bank has a limit of 75 families and some sperm donors have been used to create over 100 families [3].

Penning's paper speaks directly to the debate over whether we should limit the usage of gamete donors [4]. Pennings broadly argues against setting limits on the numbers of offspring or families and discusses not only the implications of being able to access a donor, but the implications for those who wish to become parents through gamete donation (recipients) being able to choose, where that is allowed, their donor. He states that if a limit is set low, say 25 families, it will have a significant impact on recipients, as many donors will reach that limit. And if it is higher, that might still have an impact as although it is likely there will be donors available to use in treatment, recipients’ choice over donors might be limited. As Pennings says, ‘The introduction of a worldwide family limit for all donors may have a larger impact on the recipients’ freedom of choice than on their ability to access donor treatment’ [1]. This is an interesting and often overlooked element in the debate over the use of donors. Some jurisdictions such as France do not allow a choice, whereas in the United Kingdom, recipients can choose donors, with the help of the clinics, usually based on physical matching with the recipients. Hence, in countries where donor selection is possible, limits on the use of donors could restrict recipients’ options, as there would be fewer available donors to choose from.

According to Pennings, ‘The most important ethical argument against limiting the number of families per donor globally is that this would reduce the total amount of available gametes, which would in turn increase waiting time and costs for recipients’. The problem here is limiting recipients’ options and this contravenes reproductive justice—it is unjust to restrict people's freedom to choose without a very good reason. The argument here is that recipient parents’ reproductive freedom is very important, and there should be a high burden of proof needed to restrict this freedom. This follows his reasoning on a number of issues, such as his position on donor anonymity [5]. The main ground for restricting recipient parents’ freedom, by restricting donor use, would be harm to donor‐conceived people caused by the large usage of donors. Pennings argues that we are restricting recipients’ choices for no good reason—if harm to donor‐conceived people could be established, then restrictions on recipients’ choices would be warranted—but it cannot.

In response, we argue for a precautionary approach. While it is true we do not have conclusive evidence of harm, we also do not have conclusive scientific evidence that large groups of donor‐siblings are *not* harmful. Given this state of uncertainty, it is prudent to adopt a precautionary approach and take steps to avoid the overuse of donors. As Pennings observes, there is little evidence on which to base policy on the appropriate limits to donor use. Some studies have found that donor‐conceived people find large groups of donor‐siblings distressing [6]. The responses from groups representing donor‐conceived people who contributed to our stakeholder review as part of drafting the ESHRE position paper wanted limits to donor use of around 10 families. Donor conception is an area where it is almost impossible to get conclusive evidence. Hence, rather than requiring the production of conclusive evidence of harm before restricting recipients' choices, we should require reasonable assurance of safety before permitting practices that may harm future people.

Pennings’ argument implicitly treats recipient parents’ interests (or rights) to be on an equal footing with other parties involved in donor conception, such as donor‐conceived people and donors. Therefore, in some situations donor‐conceived people's interests might not be given priority. In the ESHRE position paper, we argue that donor‐conceived people's interests should be paramount. This is not to say the interests of recipients and donors are not important, but both these groups, particularly donors, have agency and can choose whether and under what terms they wish to participate in donor conception. Further, as donor conception is an endeavour that creates children, the child's welfare should be given priority wherever possible. The recent direction of travel in donor‐conception, with the removal of anonymity, has been to see donor‐conceived people's interests as having a higher priority and that it is important to seek to safeguard these interests in the longer term throughout the donor‐concieved person's life‐course.

In sum, Pennings’ discussion of recipient choice and making sure that donation programmes operate as efficiently as possible, to both optimise access to gametes and choice over gametes, as far as that is possible and as allowed in the jurisdiction, is an important one. In flagging that more could be done to improve services and hence choice for recipients, he draws attention to flaws in existing donation programmes, and attention needs to be paid to how services could be organised more efficiently to ensure equality and equitable access to donor gametes. However, we argue that there are strong arguments for limiting the use of donors and the ESHRE position paper sets out a call for a European wide limit. This is key to ensuring that donor conception is practised ethically and that we are not creating harm for future generations by the overuse of donors.

## Author Contributions

L.F. and J.K.M. conceived on writing the response, L.F. drafted the first draft, J.K.M. rewrote and commented on it, L.F. finalised the paper. and J.K.M. approved it.

## Funding

The authors have nothing to report.

## Conflicts of Interest

The authors declare no conflicts of interest.

## Data Availability

Data sharing is not applicable to this article as no datasets were generated or analysed during the current study.
